# MicroRNAs and the Mediterranean diet: a nutri-omics perspective for lung cancer

**DOI:** 10.1186/s12967-024-05454-7

**Published:** 2024-07-07

**Authors:** Roberto Cuttano, Francesco Mazzarelli, Kuku Miriam Afanga, Fabrizio Bianchi, Elisa Dama

**Affiliations:** Unit of Cancer Biomarkers, Fondazione IRCCS Casa Sollievo della Sofferenza, Viale Cappuccini Snc, 71013 San Giovanni Rotondo, Italy

**Keywords:** Lung cancer, Prevention, Mediterranean diet, microRNA, Nutri-omics

## Abstract

Lung cancer is the deadliest cancer type worldwide with ~ 1.8 million deaths per-year. Smoking accounts for ~ 85% of all cases, with a described joint effect with unhealthy diet in lung cancer risk increase. Public health policies to prevent carcinogens exposure, promote smoking cessation and advocacy for healthy nutrition, are therefore highly recommended. Here we have examined the benefits of the Mediterranean Diet (MedDiet) in protecting against some non-communicable diseases including lung cancer, highlighting the epidemiological and biomolecular aspects of MedDiet anti-inflammatory effect and its interaction with smoking habits closely linked to risk of lung cancer. Considering the high incidence and mortality rates of lung cancer, we discussed also about the global impact that a Planeterranean extension of the benefits of MedDiet could have on controlling lung cancer risk. We also debated the impact of personalized nutrition on lung cancer prevention, considering individual heterogeneity in response to diet plans as well as recent advancements on nutri-omics in lung cancer research, with a specific focus on the role of microRNAs (miRNAs) as a promising nutritional molecular hub for lung cancer prevention. We strongly believe that a deep understanding of the molecular link between food components and genetic/epigenetics factors can expand effective intervention strategies.

## Background

Lung cancer (LC) is the leading cause of cancer deaths worldwide, with an estimated ~ 2 million new diagnoses and ~ 1.8 million deaths, every year [1]. The absence of symptoms in early-stage lung tumors pose challenges to the diagnostic process, which often results in late diagnosis when the tumor has already metastasized. Unfortunately, this gives rise to a poorer prognosis (< 32% at 5 years, [2]). More efforts are therefore needed to promote programs for LC early detection and prevention, by low-dose CT (LDCT) in high-risk individuals (aged 50–80 years, who have at least a 20 pack-years smoking history) [3, 4], to identify clinically useful biomarkers [5]. This also necessitates the adoption of public policies aimed at promoting healthier lifestyles and an awareness of cancer risk factors [6].

Cigarette smoking is the major risk factor for LC, and there is a well-established correlation between the incidence of lung cancer and the smoking policies enacted in a specific geographical area. Recent studies have revealed that more than 50% of cases are detected in developing countries, on the contrary, incidence rates are on the decline in developed countries [7]. In addition to tobacco smoking, there are many other risk factors such as personal or family history of LC, previous respiratory diseases, chest radiation, exposures to radon and other carcinogenic substances (e.g. asbestos), living in heavily polluted areas, and last but not least is the diet consumed [8]. Whilst interactions of diet with other risk factors are not yet fully investigated, literature clearly indicates a joint association of smoking and unhealthy diet in increasing the risk of lung cancer [9–12]. Particularly, eating habits were shown to be associated with risk of LC, as certain foods have been reported to offer a protective effect, while others are associated with an increased risk when consumed in excess. For example, a high consumption of vegetables, fruits, and whole grains, which characterizes the Mediterranean Diet (MedDiet) are protective against a plethora of diseases including LC [13–15]. Considering the dramatic burden that LC shows worldwide, with particular impact in developing countries, a Planeterranean extension of the benefits of MedDiet holds a global interest on LC risk control.

Here, we conducted a narrative review of the most relevant literature about the influence of dietary choices on the risk of developing LC, with a focus on the benefit of MedDiet for LC prevention, particularly in smokers. We critically analyzed recent advancements in precision nutrition and nutri-omics in LC research, with a specific focus on the role of microRNAs (miRNAs) as a promising nutritional molecular hub for exploring the interaction between food constituents and innovative approaches to prevent and treat LC.

## Methods

The PubMed database was initially queried with the following search terms “Mediterranean diet + lung cancer”, “Nutri-omics + lung cancer”, “microRNA and nutrients”. Each paper title identified from this initial search was reviewed to identify papers most pertinent to the topic. Abstracts of these papers were read, and a subset of these were selected for review of the complete manuscript. Additional relevant papers not previously identified through PubMed initial search were selected through revision of the references cited in these manuscripts. The search was limited to the English language literature of the last 30 years, with major focus on the last 15 years.

## Mediterranean diet and lung cancer

### From the origin of Mediterranean diet to the worldwide extension

MedDiet is a traditional eating pattern based on habits of the countries bordering the Mediterranean Sea. The foundation of this diet originates from the pioneer work of Ancel Keys and co-workers. They formally demonstrated low rates of coronary heart disease and all-cause mortality for people from Crete, Greece and southern Italy, who had been following food habits dated to the early 1960s [16]. The MedDiet is commonly depicted as the well-known food pyramid introduced by Walter Willett [17], with several subsequent updates [18, 19], including the recent rendition by the University of Sapienza (Italy) in the “Sapienza count-down” [20].

Although pyramids are very useful to display the general principles of the MedDiet, they include only an approximate recommendations for quantities of food groups, with major variations across several studies [18]. Frequently, high consumption of vegetables, fruits and whole grains are emphasized and positioned at the bottom of the pyramid, and extra-virgin olive oil is indicated as the main source of fat. Moreover, wine, fish, poultry, and dairy products are suggested to be consumed in low to moderate amounts, whilst minimal consumption is advised for red and processed meats. In Table 1, we reported indications on type and quantity of food required to follow MedDiet, as indicated by Sofi et al. [21]. These principles are in line with recommended dietary regulations by the World Health Organization (WHO), as summarized in Table 1 [22].

**Table 1:** Portions of food to reach the maximum adherence score to the MedDiet (adapted from Sofi et al. Public Health Nutrition, 2013 [21]), with healthy diet principles provided by the World Health Organization (adapted from: Healthy diet. Available at https://www.who.int/news-room/fact-sheets/detail/healthy-diet, accessed on Jun 2023)

	Food group	Quantity	Indication
Sofi et al. [21]	Fruits	150 g/per portion 1 apple or 1 pea or 1 orange = 150 g 3 prunes or 3 mandarins = 150 g	> 2 per-day
	Vegetables	100 g/per portion 2 tomatoes = 100 g 1 serving of salad = 70 g half-plate of cooked vegetables = 100 g	> 2.5 per-day
	Legumes	70 g/per portion Half can of beans/chickpeas/lentils = 70 g	> 2 per-week
	Cereals	130 g/per portion 1 slice of bread = 50 g 1 serving of pasta = 80 g	> 1.5 per-day
	Fish	100 g/per portion	> 2.5 per-week
	Meat and meat products	80 g/per portion 1 slice of meat = 100 g 3 slices of ham = 50 g	< 1 per-day
	Dairy products	180 g/per portion 1 cup of milk = 150 ml 1 yogurt = 125 ml 1 mozzarella cheese = 100 g	< 1 per-day
	Alcohol	1 Alcohol Unit (AU) = 12 g 1 glass of wine = 1 AU 1 can of beer = 1 AU	1–2 AU per-day
	Olive oil		Regular use (every day, no other fats)
Healthy diet [22]	Fruit and vegetables	At least 400 g per-day	Fruit and vegetable intake can be improved by: *always including vegetables in meals; *eating fresh fruit and raw vegetables as snacks; *eating fresh fruit and vegetables that are in season; *eating a variety of fruit and vegetables
	Fats	Total fat intake < 30% of TEI Saturated fats < 10% TEI *Trans*-fats < 1% TEI	Unsaturated fats are preferable to saturated fats and *trans-*fats Fat intake can be reduced by: *steaming or boiling instead of frying; *replacing butter, lard and ghee with oils *eating reduced-fat dairy foods and lean meats, or trimming visible fat from meat; *limiting baked and fried foods, and pre-packaged snacks and foods with industrially-produced *trans-*fats
	Salt, sodium and potassium	< 5 g per day Salt should be iodized	Salt intake can be reduced by: *limiting the amount of salt and high-sodium condiments when cooking and preparing foods; *not having salt or high-sodium sauces on the table; *limiting salty snacks; *choosing products with lower sodium content Potassium can mitigate the negative effects of elevated sodium consumption on blood pressure; intake of potassium can be increased by consuming fresh fruit and vegetables
	Sugar	< 10% TEI 50 g = 12 level teaspoons	Sugars intake can be reduced by: *limiting foods and drinks containing high amounts of sugars; *eating fresh fruit and raw vegetables as snacks instead of sugary snacks

Besides the indication on type and quantity of food, regular physical activity, adequate rest, and conviviality are key elements of the MedDiet. More specifically, the MedDiet is now considered not only as an eating pattern, but a cultural model and a sustainable framework [23–26] including “a set of skills, knowledge, practices and traditions ranging from the landscape to the table; it includes not only the crops but also the harvesting, fishing, conservation, processing, preparation and, particularly, consumption of food”, as stated by UNESCO who recognized the MedDiet as an Intangible Cultural Heritage of Humanity [27].

Although the MedDiet is deeply embedded in the culture of countries that border the Mediterranean sea, high-impact projects are now focusing on the adoption of the MedDiet by other populations [28], without eroding their distinct dietary, cultural, and economic habits [29, 30]. The goal of these high-impact projects is to propose a “Planeterranean” diet comprising of nutritional pyramids specific for each non-Mediterranean macro-area, and composed of foods typical of each area, while maintaining the same nutritional properties of the MedDiet and following principles of the circular economy [28, 31].

### Mediterranean diet adherence and impact on health

To effectively measure and study the adherence to MedDiet in the population, different scoring methods were applied, mostly based on self-reported questionnaires [32]. Unfortunately, these methods are typically subjective, and include limited food composition tables that do not take into account factors influencing nutrients absorption, thus providing an unreliable evaluation of food intake [33]. Therefore, reliable nutritional biomarkers are urgently needed to obtain a more objective and quantitative measure of nutrient intake [33, 34]. For instance, carotenoids and vitamin C detected in plasma or serum have been suggested as biomarkers for fruit and vegetables consumption [35], and a dose-dependent relationship between whole-grain intake and plasma alkylresorcinols has also been observed [36, 37]. These new and precise measures have potential to further reveal the extent of the established protective link between the MedDiet and some non-communicable diseases (NCDs), such as cardio-vascular disease, neurogenerative disease, type 2 diabetes and certain cancers [21, 34, 38–40]. For example, a reduced incidence of LC has been associated to a higher intake of dietary fiber from fruits, vegetables, legumes and whole-grain products [40, 41].

The largest comprehensive analysis of evidence related to the prevention and survival of cancer through control of nutrition and physical activity is conducted and periodically updated as part of the Continuous Update Project (CUP) of the World Cancer Research Fund (WCRF), and the American Institute for Cancer Research (AICR). The last update was published in 2018, with the Third Expert Report on “Diet, Nutrition, Physical Activity and Cancer: A Global Perspective” [42], confirming and giving robustness to previous findings of the CUP. According to level of evidence showed in this Third Expert Report, we summarized the role of “wholegrains, vegetables, and fruit”, vs “meat, fish, and dairy products” on the risk of cancer (Table 2), thus highlighting the impact of two food macro-categories represented in the MedDiet at high and low consumption, respectively.

**Table 2:** Impact of selected food components of MedDiet on the risk of cancer (DR = Decreased Risk; IR = Increased Risk)

Exposure	Cancer site	Effect	Level of evidence
Wholegrains, vegetables and fruit			
Wholegrains	Colorectal	DR ↓	Strong
Non-starchy vegetables and fruits (aggregated)	Aerodigestive and some other cancer	DR ↓	Strong
	Bladder	DR ↓	Limited
Non-starchy vegetables	Mouth, pharynx and larynx Nasopharynx Oesophagus (adenocarcinoma) Oesophagus (squamous cell carcinoma) Lung (smokers/former smokers) Breast (oestrogen receptor-negative)	DR ↓	Limited
	Colorectal (low intake)	IR ↑	Limited
	Nasopharynx (preserved food)	IR ↑	Limited
Fruit	Oesophagus (squamous cell carcinoma) Lung (smokers/former smokers)	DR ↓	Limited
	Stomach (low intake) Colorectum (low intake)	IR↑	Limited
Citrus fruit	Stomach (cardia)	DR ↓	Limited
Foods containing dietary fiber	Colorectal	DR ↓	Strong
Foods containing carotenoids	Lung Breast	DR ↓	Limited
Foods containing beta-carotene	Lung	DR ↓	Limited
Foods containing vitamin C	Lung (smokers) Colorectum (colon)	DR ↓	Limited
Foods containing isoflavones	Lung (never smokers)	DR ↓	Limited
Beta-carotene	Prostate	No effect	Strong
Aflatoxins	Liver	IR ↑	Strong
Foods preserved by salting	Stomach	IR ↑	Strong
Meat, fish and dairy products			
Red meat	Colorectum Nasopharynx	IR ↑	Strong
	Nasopharynx Lung Pancreas	IR ↑	Limited
Processed meat	Colorectum	IR ↑	Strong
	Nasopharynx Oesophagus (squamous cell carcinoma) Lung Stomach (non-cardia) Pancreas	IR ↑	Limited
Grilled (broiled) or barbecued (charbroiled) meat and fish	Stomach	IR ↑	Limited
Foods containing haem iron	Colorectum	IR ↑	Limited
Fish	Liver Colorectum	DR ↓	Limited
Cantonese-style salted fish	Colorectum Nasopharynx	IR ↑	Strong
Dairy products	Colorectum	DR ↓	Strong
	Breast (premenopause)	DR↓	Limited
	Prostate	IR ↑	Limited
Diets high in calcium	Breast	DR ↓	Limited
	Prostate	IR ↑	Limited

Adapted from: World Cancer Research Fund/American Institute for Cancer Research. Continuous Update Project Expert Report 2018. Diet, Nutrition, Physical Activity and Cancer: a Global Perspective. Available at dietandcancerreport.org [42]

For LC, convincing causal evidence was found for arsenic in drinking water, and high-dose consumption of beta-carotene supplements (in current and former smokers) [43–45]. Yet, limited association was observed for the consumption of red and processed meat [46, 47], and alcoholic drinks [42]. Although, limited evidence show that the consumption of vegetables, fruit, and foods containing carotenoids, beta-carotene, retinol, vitamin C and isoflavones, is associated with protective effect on LC risk (Table 3) [42].

**Table 3:** Impact of food and physical activity on lung cancer

Effect	Exposure	Level of evidence	Mechanism
Increased risk	Arsenic in drinking water	Strong	Genotoxic, chromosomal mutagenic and synergistic co-mutagenic effects; changes in the methylation of oncogenes or tumor-suppressor genes; interferes with several enzymes of the haem biosynthetic pathway
	High-dose beta-carotene supplements	Strong	Interaction with smoking and genetics, particularly in heavy smokers with genetic variation in GSTM
	Red meat	Limited	Mutagenic and carcinogenic effects of heterocyclic amines and polycyclic aromatic hydrocarbons (when cooked at high temperatures); increased production of free radicals by haem iron
	Processed meat	Limited	Mutagenic and carcinogenic effects of* N*-nitroso compounds, heterocyclic amines, and polycyclic aromatic hydrocarbons (when cooked at high temperatures); increased production of free radicals by haem and iron
	Alcoholic drinks	Limited	Carcinogenic effect of reactive metabolites (acetaldehyde); interaction with smoking; enhanced penetration of other carcinogenic molecules into mucosal cells; prostaglandins, lipid peroxidation and free radical oxygen species; interaction with diets low in essential nutrients, making tissues susceptible to carcinogenesis
Decreased risk	Non-starchy vegetables (in smokers/former smokers)	Limited	Cancer-preventive substances, including several nutrients (such as pro-vitamin A, carotenoids, and vitamin C) and dietary fibers, as well as phytochemicals (such as glucosinolates, dithiolthiones, indoles, chlorophyll, flavonoids, allylsulphides and phytoestrogens); antioxidant activity, modulation of detoxification enzymes, stimulation of the immune system, antiproliferative activity, ligand-dependent signaling through retinoid receptors and/or modulation of steroid hormone concentration and hormone metabolism; role in the synthesis and methylation of DNA
	Fruit (in smokers/former smokers)	Limited	Nutrients, such as vitamin C and a diverse array of phytochemicals, such as carotenoids, phenols, and flavonoids; flavonoids found in fruit modular cytochrome P450 enzyme systems are involved in the metabolism of carcinogens
	Foods containing retinol	Limited	Binding to a family of receptors involved in differentiation, membrane structure and function, and immunological effects associated with carcinogenesis
	Foods containing beta-carotene	Limited	Precursor of retinol; regulating host responses to oxidant stress and protection from DNA damage and the carcinogenic cascade caused by free radicals
	Foods containing carotenoids	Limited	Precursor of retinol and other metabolites with interaction with members of the steroid receptor superfamily, function in cellular differentiation, immunomodulation, and activation of carcinogen-metabolizing enzymes, protecting cells and tissues from oxidant stress and free radicals that may cause DNA damage
	Foods containing vitamin C (in smokers)	Limited	Trapping of free radicals and reactive oxygen molecules, protecting against lipid peroxidation, reducing nitrates, and stimulating the immune system; regenerative effect of vitamin E, another antioxidant vitamin; inhibition of formation of carcinogens and protection of DNA from mutagenic attack
	Foods containing isoflavones (in never smokers)	Limited	Inhibition of expression of CYP1A1, resulting in decreased formation of reactive carcinogen metabolites that form DNA adducts (primarily in smokers)
	Physical activity	Limited	Raising of the metabolic rate and increase of maximal oxygen uptake; increase of the body’s metabolic efficiency and capacity (the amount of work that it can perform), as well as reduction of blood pressure and insulin resistance

Adapted from: World Cancer Research Fund/American Institute for Cancer Research. Continuous Update Project Expert Report 2018. Diet, Nutrition, Physical Activity and Cancer: A Global Perspective. Available at dietandcancerreport.org [42]

The beneficial effect of MedDiet relies on several bioactive compounds such as polyphenols, monounsaturated and polyunsaturated fatty acids, and fiber which are associated with the reduction of blood lipids, protection against oxidative damage, improvement of insulin sensitivity, enhancement of endothelial function, and promotion of antithrombotic function [48]. Considering the pivotal role of inflammation in the etiology of LC [49], a specific dietary inflammatory index (DII) [50], that links food patterns to inflammatory markers, was investigated to elucidate the association with LC risk both in the general population [9], and in high-risk subjects enrolled in LC screening trials (i.e., aged ≥ 50 years, with a smoking history of ≥ 20 pack-years, who were current smokers or had quit smoking for < 10 years) [10]. DII was found to be inversely correlated with a MedDiet adherence score [10], while the MedDiet was described to reduce the risk of LC, especially in smokers [9, 10]. These findings confirm that the abundance of anti-inflammatory foods in the MedDiet may indeed contribute to reducing the risk of LC, particularly in heavy smokers.

Further evidence is provided by a recent meta-analysis of observational studies on the associations between diet quality and risk of lung cancer [14]; indeed, this work highlighted how high-quality diets may reduce LC risk due to higher intake of vegetables and fruits, a lower intake of animal products, and rich in antioxidant and anti-inflammatory foods. Definitely, this high-quality pattern refers not only to MedDiet but is partially shared with other forms of diet, including the Dietary Approaches to Stop Hypertension (DASH) and other plant-forward diet [14, 51–53].

Besides the association between MedDiet and LC risk, no evidence currently exists regarding the potential impacts of this dietary pattern on LC prognosis and treatment efficacy. Interestingly, a recent cohort study demonstrated a connection between adherence to MedDiet and improved progression-free survival, and objective response rate in patients receiving immune checkpoint blockade therapy for advanced melanoma [54]. Considering that the use of immune checkpoint blockade (ICB) has completely changed treatment outcomes also of LC patients [55, 56], future research is thus warranted to investigate the potential impact of the MedDiet in LC patients treatment outcomes.

## Towards nutri-omics for lung cancer

### Precision nutrition and nutri-omics

Precision nutrition has recently gained interest as a new research field with interrelated principles akin to those in precision medicine. The key paradigm of precision nutrition asserts that each individual has a different response to the same dietary exposure, which is due to inter-individual variability in the interaction between the diet consumed and genetic-metabolic-microbiome factors [57]. Therefore, there is a pressing need to tailor a personalized nutritional pattern at an individual level in order to optimize their health, prevent disease, and enhance therapeutic benefit [58]. To achieve personalized nutritional advice according to different nutritional phenotypes, the huge complexity of genetic-metabolic-microbiome and nutrition interaction should be deconvolute. Coping with this, the application of nutri-omics, i.e. an emerging science that apply omics technologies in nutritional field, has been recognized as a new powerful approach [58]. The application of high-throughput platforms for multi-omics profiling represents a powerful approach to comprehensively characterize the host and its microbiome, and their interaction with nutrition [58]. The integration of data from genomics (polymorphisms and other structural genetic variants), epigenomics (DNA methylation, histone modifications, telomere length), metagenomics (gut microbiota composition, enterotypes), transcriptomics (gene expression patterns), proteomics (protein expression and modification patterns), and metabolomics (metabolite pattern) have given rise to the identification of some potential molecular targets and active biomarkers involved in many nutritional disorders (including obesity, dyslipidemias, fatty liver, insulin resistance), inflammation, cardiovascular diseases and cancer [59]. Studies focused on the influence of nutrition on epigenetic mechanisms, such as changes (reversible) in DNA methylation and histone modifications are particularly promising for cancer prevention, since epigenetic abnormalities may occur at a very early stage during neoplastic transformation [60]. Interestingly, recent reports suggest that miRNAs (small non-coding RNAs which regulate gene and protein expression), are able to mediate the interaction between dietary regimens and a variety of molecular pathways, in both physiological and pathological conditions [61–64].

### MicroRNAs and lung cancer

MiRNAs are short non-coding RNA molecules of ~ 22 nucleotides in size which function as endogenous triggers of the mRNA interference pathway, and are involved in the regulation of many cellular processes, including differentiation, proliferation and apoptosis [65]. In the last 20 years, several studies showed that a sizable fraction of miRNAs take part either to cancer onset and progression through the activation of cancer cell-intrinsic pathways, and by triggering cancer immunoevasion processes [65]. The relevance of miRNAs in cancer is exemplified by the fact that the second ever identified miRNA, namely lethal-7 (let-7), was found to be a negative regulator of the Ras family of guanosine triphosphatases (GTPases), oncogenic in many tumor types including LC (e.g. KRAS) [66]. Following these pioneer studies, in the beginning of 2000s, many other tumor suppressor miRNAs were described such as miR-15a and miR-16-1 in B-CLL [67], and miR-34a induced by p53 [68], or oncogenic miRNAs, such as the miR-17 ~ 92 cluster (aka OncomiR-1) induced by c-Myc [69].

**Fig. 1:**
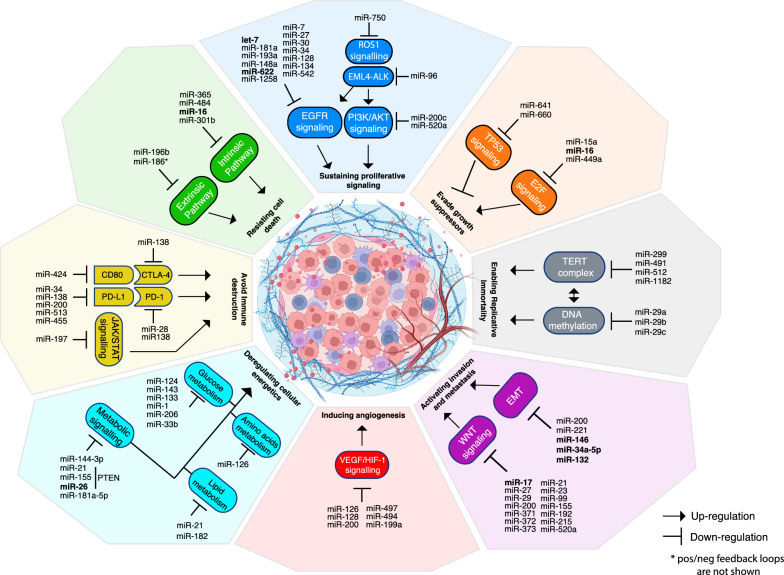
MicroRNA-mediated regulatory networks in cancer hallmarks: comprehensive map of pathways and interactions in lung cancer. The figure delineates the intricate interplay of microRNAs with the “Hallmarks of cancer” i.e. biological processes which are pivotal in tumor onset, maintenance, and progression (in bold, in the middle of the figure). The name of relevant miRNAs is reported together with their direct modulatory function (as per the legend) on the represented pathways which are listed within the colored shapes. Potential synergies and interactions between the delineated pathways can exist but were not shown in the figure. In bold, miRNA influenced by food-components (as also reported in Table 4)

Besides the role of let-7a in NSCLC [66, 70], in the last decades a sizable fraction of miRNAs were implicated in modulating LC tumor suppressor or oncogenic mechanisms [71, 72], as well as many cancer pathways including RAS, RTKs, BRAF/MAPK, PI3K, PTEN, LKB1/AMPK, TP53, RB1/MYC, JAK/STAT and Wnt/β-catenin, which impact LC growth and metabolism, tumor microenvironment, angiogenesis, tumor invasion, and metastasis [71, 72]. Comprehensive reviews have been recently proposed by Kiełbowski et al. [71] and Wang et al. [72], listing miRNAs with abnormal expression that impact on specific target genes with established role on LC. In Fig. 1, we schematically depicted the complex interaction of selected miRNAs and the hallmarks of cancer and, in particular, we showed: (i) relevant miRNAs targeting proliferative signaling i.e. ROS1 (miR-750), EML4-ALK (miR-96), PI3K/AKT (miR-200c), and EGFR signaling (let-7); (ii) miRNAs involved in LC growth suppression through regulation of TP53 (miR-660), and E2F (miR-16) regulation; (iii) miRNAs involved in replicative immortality through TERT complex (miR-299) and DNA methylation (mir-29) regulation. Moreover, other miRNAs retain a well-recognized role on invasion and metastases through WNT signaling and EMT, angiogenesis through VEGF/HIF-1 signaling, cellular metabolism, immune system, and cell death (Fig. 1).

As an additional example, miR-34a involved in p53-signalling was found to induce apoptosis in LC [73]; miR-197-5p, miR-93-5p, miR-378a-3p and miR-98-5p downregulate the expression of FUS1/TUSC2 [74], a tumor suppressor gene located on Chr.3p21.3 which is frequently hit by heterozygous (LOH) and homozygous deletions in both small (SCLC) and non-small cell lung cancers (NSCLC) [75]; oncomiR-1 was found to be upregulated in LC and to target critical genes involved in proliferation and tumor angiogenesis [76] and overall in pathogenesis of LC [77]. Interestingly, increased EGFR expression, which correlates with decreased expression of miR-128b located on Chr.3p22.3, was found coherently associated with survival benefit in gefitinib-treated patients [78].

Furthermore, miRNAs have been implicated in regulating mechanisms of chemo- and immuno-therapy resistance [79, 80] and were also investigated as accessible cancer biomarkers due to their abundance and excellent stability in body fluids [81–83]. Indeed, cells release a multitude of miRNAs in extracellular environment encapsulated in extracellular vesicles (EVs), which allow short and long-distance cell-to-cell communications [84]. One of the most relevant mechanisms is through exosomes, these are small nanosized extracellular vesicles (sEVs) of ~ 40–150 nm in diameter, that protect their enclosed miRNAs (exo-miRNAs) from degradation, and can deliver these miRNAs to other targeted cells in various (even distant) body parts. Therefore, sEVs act as stable vehicles of exo-miRNAs, which are able to function as master regulators of many cancer cellular pathways, including cell proliferation, cell differentiation, cell migration, metabolism, inflammation, angiogenesis and apoptosis (Fig. 1) [85].

### MicroRNA and MedDiet

Various dietary regimens, such as energy-controlled diets, fat-focused dietary plans, and diverse dietary patterns (e.g. vegan, vegetarian, omnivorous diets, as well as specific diets like the MedDiet) have been observed to potentially influence the expression of endogenous miRNAs within human cells. Moreover, besides unveiling the link between nutrients and endogenous miRNAs modulation, researchers have recently gained an interest in the role of food-derived miRNAs (exogenous miRNAs) and their potential implications in health and disease. In the following session, we discuss about the role of both endogenous and exogenous miRNAs on MedDiet.

#### Endogenous miRNAs and MedDiet

A diet rich in polyphenols, like the MedDiet, has been revealed to impact the expression of several miRNAs [61]. Naringenin, a polyphenolic compound found in citrus fruits, can regulate miRNAs and consequently influence gene expression profiles [61]. Similarly, other polyphenolic compounds like apigenin and ellagic acid, found in various fruits and vegetables, also demonstrate the potential to modulate miRNAs expression [61]. Concurrently, specific miRNAs like miR-25-5p, miR-148b-3p, and miR-501-3p exhibited differential expression in the plasma of mice following oral administration of flavonoids, another crucial MedDiet component [62]. In the context of LC, several food compounds have been reported to modulate endogenous miRNAs (Fig. 1). Although the exact mechanisms by which the MedDiet through miRNAs modulation impact the pathogenesis and progression of LC is currently unknown, these findings support a miRNA-mediated MedDiet protective role in the development of LC [86]. Here we summarized current evidences available in literature (Table 4).

**Table 4:** Effects of selected compounds characterizing foods of the MedDiet on miRNAs modulation and mechanisms in lung cancer

Food group	Compounds	Mirna	Mechanism	References
Vegetables and fruit, tea, wine	Apigenin	miR-34a-5p	Potential downregulation of SNAI1, inducing apoptosis	Aida et al. [89]
Vegetables and fruit, tea, wine	Apigenin	miR-21	Potential inhibition of IL-8 expression in epithelial cells exposed to cigarette smoke	Pace et al. [90]
Citrus fruits	Hesperidin	miR-132	Potential downregulation of ZEB2	Tan et al. [94]
Vegetables and fruit	Quercetin	miR-16	Downregulation of Claudin-2 expression	Sonoki et al. [95]
Vegetables and fruit	Quercetin	miR-16-5p	Downregulation of WEE1 expression, and promotion of apoptosis in radio-resistant cells	Wang et al. [96]
Vegetables and fruit	Quercetin	miR-34a-5p	Inhibition of proliferation, migration/invasion, and enhancement of apoptosis	Chai et al. [97]
Vegetables and fruit	Quercetin	let-7 family; miR-146 family; miR-26 family; miR-17 family	Reduced risk of LC	Lam et al. [86]
Grapes and red wine	Resveratrol	miR-622	Downregulation of KRAS expression, and inhibition of cancer cell proliferation	Han et al. [100]
Grapes and red wine	Resveratrol	miR-520 h	Modulation of PP2A/C-FOX2C axis, and tumor suppression activity	Yu et al. [101]
Grapes and red wine	Resveratrol	miR-671-5p	Increased sensitivity of paclitaxel resistant cells, by regulating STOML2 expression	Kong et al. [102]
Vegetables	Sulforaphane	miR-616-5p	Downregulation of GSK3b, and inhibition of EMT	Wang et al. [103]
Vegetables	Sulforaphane	miR-19	Downregulation of GSK3b, and inhibition of stem-like properties	Zhu et al. [104]
Vegetables	Sulforaphane	miR-214	Downregulation of c-Myc expression, inhibition of stem-like properties, and promotion of cisplatin cytotoxicity	Li et al. [105]
Vegetables	Sulforaphane	miR-9-3p	Potential CDH1 downregulation	Gao et al. [106]

#### Apigenin

Apigenin is a flavonoid molecule naturally occurring in numerous fruit, vegetables and beverages (e.g. chamomile, oranges, tea, and wine) [87]. It has demonstrated potential therapeutic efficacy against various cancers, especially hepatocellular carcinoma (HCC), prostate cancer, and LC, due to its anti-apoptotic, anti-proliferative, and anti-invasive properties [88]. In line with this, apigenin was identified to up-regulate the expression of miR-34a-5p in lung adenocarcinoma A549 cells in vitro, inducing apoptosis by down-regulation of SNAI1 [89]. Moreover, apigenin was able to inhibit miR-21 and IL-8 up-regulation induced by in vitro treatment of A549 with cigarette smoke extract, thus suggesting a potential role in counteracting smoke-related effects [90].

#### Hesperidin

Hesperidin, another flavonoid abundant in citrus fruit, was reported to exert anti-proliferative [91], pro-apoptotic [92] and anti-invasive [93] properties using in vitro models of LC. Accordingly, hesperidin was found to increase the levels of miR-132 in rats implanted with NSCLC cells, which in turn could regulate the expression of ZEB2, an important regulator of EMT [94].

#### Quercetin

Another flavonoid polyphenol present in fruits and vegetables of MedDiet is quercetin, which was reported to decrease the expression of Claudin-2, by upregulation of miR-16 in lung adenocarcinoma A549 cells in vitro [95]. Interestingly, miR-16-5p has been observed to be upregulated upon quercetin treatment in radiation-resistant NSCLC cell lines in vitro [96]. In this context, miR-16-5p inhibited the expression of WEE1, and increased the apoptosis rate of NSCLC cells [96]. In another study, quercetin treatment increased the expression of miR-34a-5p in vitro and this upregulation contributed to quercetin-mediated effects on proliferation, apoptosis rate and migration/invasion [97]. Importantly, Lam et al. observed that a diet with high level of quercetin, which had been previously associated with a reduced risk of LC [86], can modulate miRNAs expression within the tumor tissue of patients diagnosed with lung adenocarcinoma [86]. Interestingly, all these miRNAs have been previously reported in the literature to be involved in mechanisms of tumor metastasis, invasion, cell proliferation, and apoptosis. Notably, members of the tumor suppressor let-7 family were significantly upregulated in former smokers diagnosed with adenocarcinoma who had a higher intake of quercetin, compared to low consumers [86].

#### Resveratrol

Resveratrol, a non-flavonoid polyphenol found in MedDiet foods such as grapes and red wine, has been widely demonstrated to have anti-cancer properties in preclinical models of LC in vitro and in vivo [98]. Resveratrol treatment has been reported to reshape the miRNome of A549 cells in vitro [99]. Mechanistically, resveratrol has been demonstrated to increase miR-622 expression in human normal bronchial cells and in NCI-H460 lung cancer cell line, in vitro [100]. Interestingly, the upregulation of miR-622 induced by resveratrol was found to downregulate KRAS (a potent oncogene in lung cancer) expression, and partially inhibit cancer cell proliferation [100]. In other studies, resveratrol treatment was shown to decrease miR-520 h expression in A549 lung adenocarcinoma cell line, which may play a role in the regulation of certain resveratrol-mediated tumor suppression activity by modulating PP2A/C-FOX2C axis [101]. Notably, Kong et al. reported that resveratrol treatment increased the sensitivity of paclitaxel resistant cells via miR-671-5p upregulation, that in turn regulates the expression of STOML2 [102].

#### Sulforaphane

Sulforaphane, an isothiocyanate molecule derived from broccoli and other cruciferous vegetables has been demonstrated to have anti-cancer properties in LC in vitro and in vivo [98]. In line with this, sulforaphane has been found to decrease the expression of miR-616-5p, thus restraining epithelial-mesenchymal transition (EMT), and the metastasis of lung cancer through the miR-616-5p/GSK3β/β-catenin signaling pathway [103]. Similarly, sulforaphane treatment inhibited GSK3β and the stem-like properties of lung cancer cells in vitro, by reducing the expression of miR-19 [104]. Additionally, other miRNAs such as miR-9-3p and miR-214 have been reported to be upregulated in vitro following sulforaphane treatment [105, 106]. Notably, Li et al. observed that miR-214 directly targets C-Myc expression and is implicated in the regulation of stem-like properties, and cisplatin resistance in the NCI-H460 cell line [105]. Interestingly, several studies conducted in humans have suggested an association between the consumption of cruciferous vegetables and a reduced risk of tobacco-related lung cancer [98]. Indeed, ongoing clinical trials are now investigating the effects of sulforaphane treatment on prevention of lung cancer (ClinicalTrials.gov: NCT03232138).

#### Exogenous food-derived miRNAs and MedDiet

The role of food-derived miRNAs and their potential implications in health and disease has been recently investigated. Exogenous miRNAs are naturally present in food and can be up-taken via the consumption of plant and animal sources, thus potentially influencing host gene expression [107]. Interestingly, exogenous miRNAs can be absorbed at the level of gastrointestinal tract and then released into the bloodstream in a naked form, associated with proteins or encapsulated in sEVs, thus reaching distant organs and affecting their healthy state [108]. A comprehensive study entitled "Food derived microRNAs" explores this subject in great depth, shedding light on how these miRNAs could mediate post-transcriptional changes in gene expression, affecting cellular processes, and potentially influencing the development of conditions such as obesity, diabetes, neurodegenerative diseases, and cancer [63]. Interestingly, these dietary miRNAs appear to maintain their functionality across different species due to their evolutionary conservation. This implies their ability to retain their regulatory capacity even when consumed and transferred from one species to another, thus potentially influencing gene expression in the consumer. For instance, plant-derived miRNAs like the strawberry fv-MIR168 and the cabbage bol-MIR874, or those from Carica papaya, such as cpa-MIR1403 and cpa-MIR0016, have been found to interact with human genes and play immunomodulatory roles. This is probably due to their 3’OH methylation of these plant-derived miRNAs, affecting T cell proliferation and apoptosis mechanisms in cancer, similarly to human miR-34a [109, 110]. In vitro and in vivo studies have shown that pt-miR-159, particularly abundant in broccoli, holds the potential to inhibit the growth of breast cancer cells by targeting TCF7-MYC axis [111]. Pt-miR-156a, which is present in a variety of vegetables consumed with MedDiet (e.g. cabbage, spinach, and lettuce), was reported to target junctional adhesion molecule A (JAM-A) and to inhibit monocyte adhesion during inflammatory stress [112]. Another interesting paper about cross-kingdom regulation demonstrated that shrimp-derived miR-34, displays a dual role: (i) it suppresses viral infection in shrimp by targeting viral genes wsv330 and wsv359; and (ii) when consumed by humans, it can inhibit the progression of diseases like breast cancer, by targeting human genes such as MET and CDK6 [113]. However, other studies actually challenged the role of dietary miRNAs as modulators of biological functions in the recipient organisms [114].

Indeed, sequencing contamination or other artifacts could produce biased results about the role of dietary miRNAs in the human body [115]. Yet, alternative evidence indicates that only minimal amounts of dietary miRNAs were recovered after the digestion process [116]. Notwithstanding, the fascinating concept of cross-species miRNA-based gene regulation has set the stage for further investigation on the utilization of nutritional strategies to enhance the efficacy of current therapeutics, and to aid the development of new ones.

##### Food-derived small extracellular vesicles

Recent evidences have proposed that small extracellular vesicles (sEVs) are present in a variety of foods (e.g., plants, vegetables, fruits, honey etc.), and are rich in bioactive compounds including mRNA, proteins, lipids, metabolites, miRNAs, and long non-coding RNAs, that are protected from degradation during digestion [64, 117, 118]. Definitely, it has been demonstrated that functional protein in the target cells can be produced from translation of mRNA encapsulated in sEVs; moreover, the bioactivity of miRNAs and proteins are preserved in the target cells [118]. Remarkably, the protected cargo can act both on cells of the gastrointestinal tract, and cells in other district of the body reached through the bloodstream, and also interact with endogenous exosomes. All these peculiarities confer to food-derived sEVs the potential of having a major role on regulation of physiological and disease condition, including cancer [118].

Indeed, recent evidences highlighted that sEVs from several plants characterizing the MedDiet, such as lemon, orange and grapefruit, are enriched in phospholipids, including phosphatidylcholine, phosphatidic acid, phosphatidylethanolamine, phosphatidylinositol, and phosphatidylglycerol [119]. Moreover, more than 1018 proteins were identified from clementine-derived sEVs, including 62 proteins under the category of Gene Ontology, counting transmembrane transport-related, vesicle-mediated, and intracellular transporters [120]. MiRNAs content in food-derived sEVs has been more investigated in the recent years. Interestingly, small RNA-seq revealed the presence of miRNAs encapsulated in plant derived exosome like nanoparticles (ELNs) from eleven different edible fruits including ones belonging to MedDiet (e.g. blueberry, grapefruit, orange, pea, pear, and tomato) [121]. In this line of thought, pt-miRNAs in ELNs were shown to regulate gene expression, modulate intestinal permeability, and influence the composition and function of gut microbiota, ultimately impacting diseases like colitis and cancer [64, 122]. Furthermore, pt-miRNA were involved in immune responses modulation by inducing the production of the cytokine IL-22, an immune protein critical for gut homeostasis and defense against pathogenic bacteria [64]. ELNs and other similar extracellular vesicles, such as honey-derived vesicle-like nanoparticles (VLNs) and plant-derived nanovesicles (PNVs), are part of a common mechanism for transport and delivery of miRNAs. Notably, honey-derived miRNAs like miR-4057 within VLNs suppress the NLRP3 inflammasome, easing inflammation and liver damage in mice [123]. PNVs, carrying miRNAs like MIR159a, MIR167a, and MIR166a, demonstrate anti-inflammatory effects, making them promising carriers for drug delivery [61].

It is imperative to note that this complex web of interactions is further influenced by cooking. Exosomes derived from cooked pork, for instance, carrying liver pork-derived miR-122, can induce insulin resistance and metabolic disorder in the liver by affecting the PPAR signaling pathway [124]. Together, these studies highlight how diet can influence human health by either introducing exogenous food-derived miRNAs into our body, or by altering endogenous miRNAs expression. From targeting specific disease genes, to modulating our microbiota and immune response, the pervasive effects of dietary miRNAs, and their relationships with extracellular vesicles, offer promising potential for their use as functional food components and therapeutic agents. Therefore, we envision that conducting a global screening of miRNAs across various food classes holds significant promise for shaping optimal dietary regimens tailored to address specific pathologies, like LC and improve treatment response.

## Discussion

LC prevention has been historically linked to anti-smoking campaigns due to the causal effect of tobacco smoking on LC development. Tobacco smoke contains hundreds of chemicals which included ~ 80 carcinogens such as polycyclic aromatic hydrocarbons (PAH) and N-nitrosamines (TSNAs). Beyond the direct mutagenic effects of these chemicals, tobacco smoke induces a high oxidative stress in lung epithelial and stromal cells which, in turn, favor the onset of chronic inflammatory processes ultimately contributing to LC development [125–127].

On the contrary, healthy dietary habits have been reported to play a significant role in LC prevention due to several anti-inflammatory and antioxidant components of specific nutrients. Above all, the MedDiet which is rich in polyphenols, fiber, and vitamins, correlates with a reduction of LC risk by dampening oxidative stress and combating inflammatory processes. Research has shown that 30–50% of all cancers are preventable thorough an adequate diet and physical activity. A global extension of MedDiet in a Planeterranean perspective could enhance the impact of diet on LC burden.

In line with this, precision nutrition is becoming an important field of research toward the identification of genomic, transcriptomics, and proteomics features, which characterize inter-individual variability to dietary exposure, and define the landscape of digested food molecules (including exogenous miRNAs and exosome-like nanoparticles) by using nutri-omics approaches. These data will be fundamental for developing accurate molecular biomarkers to predict gene-nutrient, nutrient-nutrient, and drug-nutrient interactions in our body. Therefore, biomarkers of nutrients exposure and nutritional status holds great promise to define personalized nutritional patterns which can prevent LC and improve therapeutic strategies.

In the present review, we proposed a new perspective on the impact of MedDiet may have in the modulation of miRNA-based molecular mechanisms involved in LC. Lung cancer risk is influenced by environmental and lifestyle factors including nutritional patterns such as MedDiet. Conversely, miRNAs which are involved in LC cancer initiation and progression can be influenced in their expression patterns by nutrients. To the best of our knowledge, a cross-discussion about the role of miRNAs in nutri-omics LC research is currently lacking which prompted us to further explore such fascinating topic. Our findings during literature review revealed that nutrients can modulate human cells endogenous miRNAs as well as release exogenous (food-derived) miRNAs which concur to influence LC risk. However, our work is limited by the lack in literature of in-depth studies on miRNA-nutrients interaction with single or combined form of diets and their relative impact on LC risk. Additionally, in order to provide a new perspective on LC risk, in the present article we focused on a very broad topic, trying to connect evidences from different disciplines ranging from LC epidemiology, nutrition and omics science. Certainly, the narrative approach we chose and the qualitative summary of the findings we provided did not allow to reach the power of systematic reviews. Future studies in this research field are warranted which could provide valuable information on new dietary interventions for the prevention and management of lung cancer.

## Data Availability

Data sharing is not applicable to this article as no new data were created in this study.

## Abbreviations

AICR: American Institute for Cancer Research
AU: Alcohol unit
CUP: Continuous update project
DII: Dietary inflammatory index
ELN: Exosome-like nanoparticle
EMT: Epithelial-mesenchymal transition
EV: Extracellular vesicles
ICB: Immune checkpoint blockade
LC: Lung cancer
LDCT: Low-dose computed tomography
MedDiet: Mediterranean diet
miRNA: MicroRNA
NCD: Non-communicable disease
PAH: Polycyclic aromatic hydrocarbons
PNV: Plant-derived nanovesicle
pt-miRNA: Plant-derived miRNA
TSNA: Tobacco-specific N-nitrosamines
VLN: Vesicle-like nanoparticle
WCRF: World Cancer Research Fund
WHO: World Health Organization
